# Predictive markers in cancer patient diagnosis, classification and prediction of therapy outcome using leukaemia as a model

**DOI:** 10.1186/1878-5085-5-S1-A26

**Published:** 2014-02-11

**Authors:** Godfrey Grech, Shawn Baldacchino, Christian Saliba, Anthony Fenech

**Affiliations:** 1 Department of Pathology, University of Malta, Malta, Italy; 2 Department of Pharmacology and Clinical Therapeutics, University of Malta, Malta, Italy

## Introduction

The characterisation of the molecular mechanism of disease allows classification of patients into subtypes and potentially identifies specific targets for therapeutic intervention. Tyrosine kinase mutations are central to specific targeted therapy. Investigation of kinase deregulation within particular patient groups, has led to identification of mutant tyrosine kinases associated with disease progression and therapy modulation. Biomarker-specific therapies emerged, taking a leading role in guided-therapy. The extensive use of the specific kinase inhibitors and the longevity of the treatment protocols due to the uncertainty of residual disease, gave rise to new challenges, namely secondary resistance to therapy. Although there are various mechanisms of acquired resistance, mutations in the drug target itself play a dominant role.

## Scientific objectives

Our previous studies using cellular models show the importance of suppressed feedback mechanisms, in particular the regulation of the phosphatase, pp2a. Following extensive molecular classification of patients, the aim of this study was to identify variants and transcript isoforms of PPP2CA and its inhibiting subunits ALPHA4 and SET, using (1) cell lines derived from haematopoietic disease, and (2) Chronic Myeloid Leukemia (CML) and Acute Myeloid Leukemia (AML) patient material.

## Technological approaches

Patients were classified according to molecular defects using well established protocols. The expression of phosphatase regulators was measured using Real Time PCR. The catalytic subunit, PPP2CA was screened for variation at the level of mutations and isoforms using High Resolution Melting analysis. cDNA was utilised for this screen. Variants were further characterised by sequencing.

## Results interpretation

A preliminary analysis investigating the co-occurrence of FLT3 and Core Binding Factor (CBF) mutations characterised these mutations simultaneously in some patients. This indicates an interaction between these two distinct classes of mutations, in addition to the well known association of KIT mutants in CBF patients. The presence of KIT mutations predicts the use of the Tyrosine Kinase Inhibitor, Imatinib. Following classifications using known mutations in kinases and transcription factors, the variation in the tumour suppressor, pp2a was investigated. Variation in the PPP2CA transcript is minimal and the identified nucleotide changes were synonymous. HRM identified mutants in SET and alpha4. Of interest the cell line, U937 has multiple mutations in the coding region of SET. PPP2CA isoforms were identified in patient material. One of the isoforms, is predominantly expressed in 15% of CML (n=19) and AML (n=344) patients. In addition, the characterisation of a novel PPP2CA isoform in the BCR/ABL positive cell line BV173 indicates that such isoforms may be associated with progression of disease and potentially chemo-resistance.

## Outlook and expert recommendations

Loss-of-function mutations in the phosphatase, pp2a and/or an enhanced pp2a inhibition due to increased expression of pp2a regulators (such as CIP2A, SETBP1) will provide a new classification of patients in various malignancies. Of interest, these subtypes will provide the basis to investigate the use of pp2a activators as therapeutic drugs.

